# (*E*)-Ethyl 2-cyano-3-(2,4-dimeth­oxy­phen­yl)prop-2-enoate

**DOI:** 10.1107/S1600536811040013

**Published:** 2011-10-05

**Authors:** Antar A. Abdelhamid, Shaaban K. Mohamed, Ali N. Khalilov, Atash V. Gurbanov, Seik Weng Ng

**Affiliations:** a Department of Organic Chemistry, Baku State University, Baku, Azerbaijan; b School of Biology, Chemistry and Material Science, Manchester Metropolitan University, Manchester, UK; c Department of Chemistry, University of Malaya, 50603 Kuala Lumpur, Malaysia; d Chemistry Department, Faculty of Science, King Abdulaziz University, PO Box 80203 Jeddah, Saudi Arabia

## Abstract

The C=C bond in the title compound, C_14_H_15_NO_4_, is in an *E* configuration. With the exception of the methyl C atoms, the non-H atoms of the mol­ecule all lie approximately on a plane (r.m.s. deviation = 0.096 Å). π–π stacking is observed between parallel benzene rings of adjacent mol­ecules, the centroid–centroid distance being 3.7924 (8) Å.

## Related literature

For benzyl­idene­cyano­acetate, see: Bodrikov *et al.* (1992 ▶) and for 3,4-dimeth­oxy­benzyl­idene­cyano­acetate, see: Nesterov *et al.* (2001 ▶).
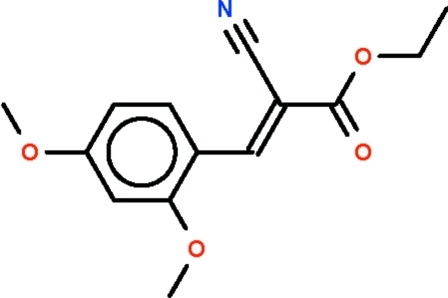


## Experimental

### 

#### Crystal data

C_14_H_15_NO_4_
*M* *_r_* = 261.27
Monoclinic, 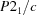

*a* = 10.5661 (6) Å
*b* = 6.9715 (4) Å
*c* = 18.4141 (10) Å
β = 101.858 (1)°
*V* = 1327.47 (13) Å^3^
*Z* = 4
Mo *K*α radiation
μ = 0.10 mm^−1^
*T* = 295 K
0.20 × 0.20 × 0.20 mm

#### Data collection

Bruker SMART APEXII diffractometer
14924 measured reflections
3330 independent reflections
2382 reflections with *I* > 2σ(*I*)
*R*_int_ = 0.026

#### Refinement

*R*[*F* ^2^ > 2σ(*F* ^2^)] = 0.046
*wR*(*F* ^2^) = 0.134
*S* = 1.03
3330 reflections
172 parameters
H-atom parameters constrained
Δρ_max_ = 0.23 e Å^−3^
Δρ_min_ = −0.21 e Å^−3^

### 

Data collection: *APEX2* (Bruker, 2005 ▶); cell refinement: *SAINT* (Bruker, 2005 ▶); data reduction: *SAINT*; program(s) used to solve structure: *SHELXS97* (Sheldrick, 2008 ▶); program(s) used to refine structure: *SHELXL97* (Sheldrick, 2008 ▶); molecular graphics: *X-SEED* (Barbour, 2001 ▶); software used to prepare material for publication: *publCIF* (Westrip, 2010 ▶).

## Supplementary Material

Crystal structure: contains datablock(s) global, I. DOI: 10.1107/S1600536811040013/xu5338sup1.cif

Structure factors: contains datablock(s) I. DOI: 10.1107/S1600536811040013/xu5338Isup2.hkl

Supplementary material file. DOI: 10.1107/S1600536811040013/xu5338Isup3.cml

Additional supplementary materials:  crystallographic information; 3D view; checkCIF report

## Figures and Tables

**Fig. 1.**
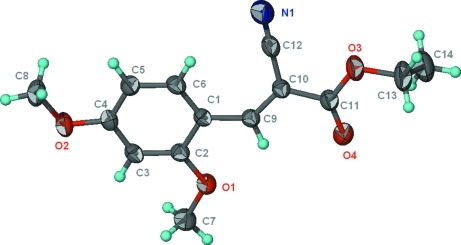
Thermal ellipsoid plot (Barbour, 2001) of C14H15NO4 at the 50% probability level; hydrogen atoms are drawn as spheres of arbitrary radius.

## supplementary crystallographic information

### Comment

The synthesis of benzylidenecyanoacetate was reported by Bodrikov *et al.* in 1992; the compound was synthesized by a conventional route. In the present study, microwave radiation was used to initiate the condensation; 2,4-dimethoxylbenzaldehyde was used in place of the unsubstituted homolog. The carbon-carbon double-bond in C_14_H_15_NO_4_ is of an *E*-configuration (Scheme I, Fig. 1). With the exception of the methyl C, the non-hydrogen atoms all lie on a plane. The features are similar to those of 3,4-dimethoxybenzylidenecyanoacetate (Bodrikov *et al.*, 1992).

### Experimental

2,4-Dimethoxy benzaldehyde (10 mmol), ethyl cyanoacetate (10 mmol), and 2,4-pentanedione (100 mmol, aprox. 10 ml) dissolved in ethanol (50 ml) and the solution was irradiated by microwave irradiation for 5 minutes. The mixture was cooled and the product was recrystalized from ethanol in 90% yield; m.p. 405 K.

### Refinement

Carbon-bound H-atoms were placed in calculated positions [C–H 0.93 to 0.97 Å; *U*(H) 1.2 to 1.5*U*(C)] and were included in the refinement in the riding model approximation, with *U*(H) set to 1.2 to 1.5*U*(C).

### Crystal data

**Table:**

C_14_H_15_NO_4_	*F*(000) = 552
*M**_r_* = 261.27	*D*_x_ = 1.307 Mg m^−^^3^
Monoclinic, *P*2_1_/*c*	Mo *K*α radiation, λ = 0.71073 Å
Hall symbol: -P 2ybc	Cell parameters from 3565 reflections
*a* = 10.5661 (6) Å	θ = 2.3–27.7°
*b* = 6.9715 (4) Å	µ = 0.10 mm^−^^1^
*c* = 18.4141 (10) Å	*T* = 295 K
β = 101.858 (1)°	Prism, colorless
*V* = 1327.47 (13) Å^3^	0.20 × 0.20 × 0.20 mm
*Z* = 4	

### Data collection

**Table:**

Bruker SMART APEXII diffractometer	2382 reflections with *I* > 2σ(*I*)
Radiation source: fine-focus sealed tube	*R*_int_ = 0.026
graphite	θ_max_ = 28.4°, θ_min_ = 2.0°
φ and ω scans	*h* = −14→14
14924 measured reflections	*k* = −9→9
3330 independent reflections	*l* = −24→24

### Refinement

**Table:**

Refinement on *F*^2^	Primary atom site location: structure-invariant direct methods
Least-squares matrix: full	Secondary atom site location: difference Fourier map
*R*[*F*^2^ > 2σ(*F*^2^)] = 0.046	Hydrogen site location: inferred from neighbouring sites
*wR*(*F*^2^) = 0.134	H-atom parameters constrained
*S* = 1.03	*w* = 1/[σ^2^(*F*_o_^2^) + (0.0663*P*)^2^ + 0.2366*P*] where *P* = (*F*_o_^2^ + 2*F*_c_^2^)/3
3330 reflections	(Δ/σ)_max_ = 0.001
172 parameters	Δρ_max_ = 0.23 e Å^−^^3^
0 restraints	Δρ_min_ = −0.21 e Å^−^^3^

### Fractional atomic coordinates and isotropic or equivalent isotropic displacement parameters (Å^2^)

**Table:**

	*x*	*y*	*z*	*U*_iso_*/*U*_eq_	
O1	0.31913 (10)	0.27747 (18)	0.55366 (6)	0.0536 (3)	
O2	0.29391 (10)	0.12247 (17)	0.30174 (6)	0.0537 (3)	
O3	0.87131 (11)	0.3785 (2)	0.71774 (6)	0.0628 (4)	
O4	0.66774 (11)	0.4110 (2)	0.73311 (6)	0.0627 (3)	
N1	0.89121 (15)	0.2321 (3)	0.55058 (9)	0.0804 (6)	
C1	0.51259 (12)	0.25722 (18)	0.50966 (7)	0.0351 (3)	
C2	0.37586 (13)	0.2438 (2)	0.49505 (8)	0.0377 (3)	
C3	0.30758 (13)	0.1990 (2)	0.42512 (8)	0.0411 (3)	
H3	0.2179	0.1899	0.4163	0.049*	
C4	0.37162 (14)	0.1674 (2)	0.36783 (8)	0.0401 (3)	
C5	0.50565 (14)	0.1815 (2)	0.37981 (8)	0.0434 (3)	
H5	0.5489	0.1616	0.3413	0.052*	
C6	0.57268 (13)	0.2257 (2)	0.45012 (8)	0.0417 (3)	
H6	0.6623	0.2350	0.4582	0.050*	
C7	0.18101 (15)	0.2627 (3)	0.54175 (10)	0.0631 (5)	
H7A	0.1535	0.2895	0.5873	0.095*	
H7B	0.1548	0.1353	0.5254	0.095*	
H7C	0.1422	0.3535	0.5046	0.095*	
C8	0.35279 (19)	0.0981 (3)	0.23941 (9)	0.0636 (5)	
H8A	0.2877	0.0668	0.1965	0.095*	
H8B	0.4151	−0.0038	0.2492	0.095*	
H8C	0.3953	0.2150	0.2305	0.095*	
C9	0.57905 (13)	0.30444 (19)	0.58405 (7)	0.0371 (3)	
H9	0.5242	0.3340	0.6160	0.045*	
C10	0.70570 (13)	0.3137 (2)	0.61586 (8)	0.0383 (3)	
C11	0.74281 (14)	0.3727 (2)	0.69485 (8)	0.0446 (3)	
C12	0.80883 (14)	0.2688 (2)	0.57949 (8)	0.0497 (4)	
C13	0.92092 (19)	0.4426 (4)	0.79351 (10)	0.0773 (6)	
H13A	0.8687	0.5488	0.8047	0.093*	
H13B	1.0088	0.4886	0.7977	0.093*	
C14	0.9198 (2)	0.2881 (4)	0.84808 (12)	0.0922 (8)	
H14A	0.9534	0.3361	0.8971	0.138*	
H14B	0.9725	0.1835	0.8378	0.138*	
H14C	0.8327	0.2442	0.8449	0.138*	

### Atomic displacement parameters (Å^2^)

**Table:**

	*U*^11^	*U*^22^	*U*^33^	*U*^12^	*U*^13^	*U*^23^
O1	0.0315 (5)	0.0867 (8)	0.0439 (6)	−0.0039 (5)	0.0112 (4)	−0.0102 (5)
O2	0.0473 (6)	0.0751 (8)	0.0352 (5)	−0.0065 (5)	0.0001 (4)	0.0012 (5)
O3	0.0433 (6)	0.0943 (10)	0.0461 (6)	−0.0115 (6)	−0.0018 (5)	−0.0126 (6)
O4	0.0539 (7)	0.0885 (9)	0.0455 (6)	0.0063 (6)	0.0098 (5)	−0.0101 (6)
N1	0.0359 (7)	0.1402 (17)	0.0662 (10)	−0.0018 (9)	0.0134 (7)	−0.0185 (10)
C1	0.0305 (6)	0.0374 (7)	0.0368 (7)	−0.0007 (5)	0.0054 (5)	0.0033 (5)
C2	0.0339 (7)	0.0416 (7)	0.0385 (7)	0.0003 (5)	0.0095 (5)	0.0027 (6)
C3	0.0296 (6)	0.0488 (8)	0.0432 (7)	−0.0020 (6)	0.0036 (6)	0.0044 (6)
C4	0.0413 (7)	0.0416 (7)	0.0348 (7)	−0.0021 (6)	0.0020 (5)	0.0049 (6)
C5	0.0408 (7)	0.0549 (9)	0.0361 (7)	0.0000 (6)	0.0117 (6)	0.0035 (6)
C6	0.0301 (7)	0.0525 (8)	0.0425 (7)	−0.0001 (6)	0.0076 (6)	0.0035 (6)
C7	0.0323 (8)	0.1026 (15)	0.0569 (10)	−0.0057 (8)	0.0149 (7)	−0.0097 (10)
C8	0.0674 (11)	0.0851 (13)	0.0361 (8)	−0.0066 (9)	0.0059 (7)	−0.0056 (8)
C9	0.0342 (7)	0.0405 (7)	0.0371 (7)	0.0004 (5)	0.0081 (5)	0.0013 (6)
C10	0.0336 (7)	0.0421 (7)	0.0387 (7)	−0.0025 (6)	0.0065 (5)	0.0004 (6)
C11	0.0398 (8)	0.0509 (9)	0.0412 (8)	−0.0030 (6)	0.0041 (6)	−0.0011 (6)
C12	0.0329 (7)	0.0704 (11)	0.0432 (8)	−0.0058 (7)	0.0017 (6)	−0.0036 (7)
C13	0.0638 (12)	0.1100 (17)	0.0506 (10)	−0.0214 (11)	−0.0059 (8)	−0.0201 (11)
C14	0.0774 (15)	0.134 (2)	0.0547 (11)	−0.0001 (14)	−0.0103 (10)	−0.0037 (13)

### Geometric parameters (Å, °)

**Table:**

O1—C2	1.3584 (16)	C6—H6	0.9300
O1—C7	1.4342 (18)	C7—H7A	0.9600
O2—C4	1.3573 (17)	C7—H7B	0.9600
O2—C8	1.4238 (19)	C7—H7C	0.9600
O3—C11	1.3370 (18)	C8—H8A	0.9600
O3—C13	1.456 (2)	C8—H8B	0.9600
O4—C11	1.1945 (18)	C8—H8C	0.9600
N1—C12	1.139 (2)	C9—C10	1.3475 (19)
C1—C6	1.3925 (19)	C9—H9	0.9300
C1—C2	1.4172 (18)	C10—C12	1.426 (2)
C1—C9	1.4431 (19)	C10—C11	1.485 (2)
C2—C3	1.376 (2)	C13—C14	1.475 (3)
C3—C4	1.383 (2)	C13—H13A	0.9700
C3—H3	0.9300	C13—H13B	0.9700
C4—C5	1.391 (2)	C14—H14A	0.9600
C5—C6	1.377 (2)	C14—H14B	0.9600
C5—H5	0.9300	C14—H14C	0.9600
			
C2—O1—C7	117.86 (12)	O2—C8—H8B	109.5
C4—O2—C8	117.79 (12)	H8A—C8—H8B	109.5
C11—O3—C13	116.96 (13)	O2—C8—H8C	109.5
C6—C1—C2	116.87 (12)	H8A—C8—H8C	109.5
C6—C1—C9	124.86 (12)	H8B—C8—H8C	109.5
C2—C1—C9	118.26 (12)	C10—C9—C1	132.04 (13)
O1—C2—C3	123.40 (12)	C10—C9—H9	114.0
O1—C2—C1	115.90 (12)	C1—C9—H9	114.0
C3—C2—C1	120.70 (12)	C9—C10—C12	124.87 (13)
C2—C3—C4	120.35 (12)	C9—C10—C11	118.55 (12)
C2—C3—H3	119.8	C12—C10—C11	116.57 (12)
C4—C3—H3	119.8	O4—C11—O3	124.19 (14)
O2—C4—C3	114.83 (12)	O4—C11—C10	124.48 (14)
O2—C4—C5	124.54 (13)	O3—C11—C10	111.33 (12)
C3—C4—C5	120.63 (13)	N1—C12—C10	179.7 (2)
C6—C5—C4	118.36 (13)	O3—C13—C14	112.15 (18)
C6—C5—H5	120.8	O3—C13—H13A	109.2
C4—C5—H5	120.8	C14—C13—H13A	109.2
C5—C6—C1	123.09 (13)	O3—C13—H13B	109.2
C5—C6—H6	118.5	C14—C13—H13B	109.2
C1—C6—H6	118.5	H13A—C13—H13B	107.9
O1—C7—H7A	109.5	C13—C14—H14A	109.5
O1—C7—H7B	109.5	C13—C14—H14B	109.5
H7A—C7—H7B	109.5	H14A—C14—H14B	109.5
O1—C7—H7C	109.5	C13—C14—H14C	109.5
H7A—C7—H7C	109.5	H14A—C14—H14C	109.5
H7B—C7—H7C	109.5	H14B—C14—H14C	109.5
O2—C8—H8A	109.5		
			
C7—O1—C2—C3	0.9 (2)	C4—C5—C6—C1	−0.1 (2)
C7—O1—C2—C1	−179.11 (14)	C2—C1—C6—C5	−0.6 (2)
C6—C1—C2—O1	−179.18 (12)	C9—C1—C6—C5	−179.78 (13)
C9—C1—C2—O1	0.05 (19)	C6—C1—C9—C10	−6.4 (2)
C6—C1—C2—C3	0.8 (2)	C2—C1—C9—C10	174.47 (15)
C9—C1—C2—C3	−179.93 (13)	C1—C9—C10—C12	−2.2 (3)
O1—C2—C3—C4	179.66 (13)	C1—C9—C10—C11	178.48 (14)
C1—C2—C3—C4	−0.4 (2)	C13—O3—C11—O4	−2.3 (3)
C8—O2—C4—C3	176.73 (14)	C13—O3—C11—C10	177.39 (15)
C8—O2—C4—C5	−3.5 (2)	C9—C10—C11—O4	0.9 (2)
C2—C3—C4—O2	179.36 (13)	C12—C10—C11—O4	−178.46 (16)
C2—C3—C4—C5	−0.4 (2)	C9—C10—C11—O3	−178.79 (14)
O2—C4—C5—C6	−179.10 (14)	C12—C10—C11—O3	1.84 (19)
C3—C4—C5—C6	0.6 (2)	C11—O3—C13—C14	81.1 (2)
